# Supplementary material for: Development of guidelines for tertiary education institutions to assist them in supporting students with a mental illness: a Delphi consensus study with Australian professionals and consumers
Source: PeerJ. 2013 Feb 26;1:e43. doi: 10.7717/peerj.43 (PMC3629038; doi:10.7717/peerj.43)
Supplement: Supplemental Figure 1 [file peerj-01-43-s002.pdf]

Support from teaching staff

- Teaching staff should let their students know that they can be approached if the students have any problems impacting on their study and that they will be supportive.
- If teaching staff are unsure of how to assist a student with a mental illness or whether the required supports are available, they should consult with the relevant support services staff.
- Teaching staff should not discuss their observations about students' potential mental illnesses in front of a lecture or tutorial group.
- Teaching staff should make the adjustments recommended by the support services for students with a mental illness.
- Teaching staff should encourage students in the helping professions and courses to increase awareness of mental illness and encourage the appropriate use of available mental health services (e.g. through setting appropriate class projects).

STUDENTS

Student rights and responsibilities

- The institution should make the students aware of their rights and responsibilities in relation to their mental illness and its effect on their studies.
- In relation to their mental illness, student's rights should include:
  1. Being in an institution that observes the principles of equal opportunity.
  2. Confidentiality of information about their mental illness, within the limits of safety.
  3. Reasonable access to support services.
  4. Being made aware of appropriate appeals procedures.
  5. Access to reasonable adjustments where necessary (e.g. extended time for exams).
- In relation to their mental illness, students' responsibilities should include:
  1. Letting needs be known to appropriate teaching and support services staff.
  2. Participating in discussions and cooperating in the process of determining special considerations.
  3. Fulfilling the academic requirements of the course.
  4. Completing appropriate administrative procedures.

Mental illness awareness

- The institution should have procedures for making students aware of the following:
  1. What mental illness is.
  2. How common mental illnesses are in Australian students.
  3. The types of mental illnesses.
  4. The warning signs and symptoms of mental illnesses.
  5. The mental health and disability support services available on-campus and in the community.
  6. The importance of early identification and intervention for young people who may be developing a mental illness.
  7. Measures that can be taken to ensure optimum mental health and prevent mental illness (e.g. self help strategies, seeing a health professional).
  8. How to help fellow students who show signs of mental illness to access support services or other mental health resources quickly.
  9. How they can support fellow students with a mental illness in ways that promote recovery.
  10. That the level of support needed by students with a mental illness will fluctuate, as the symptoms of most mental illnesses come and go over time.
  11. The impact of the symptoms of mental illness on the skills necessary for work and study, such as problems with concentration, memory, decision making and motivation.
  12. The pressures of student life and the stresses involved with study, including the amount of work expected, to allow them to make decisions about realistic study loads, particularly if they have a mental illness.
  13. The things they may notice which might indicate that a student has a mental illness, such as effects on attendance, handing in assignments, displaying unusual behaviours.
  14. The benefits of disclosing their mental illness to the institution (e.g. to allow access to support services).
  15. The myths surrounding mental illness which lead to stigma and limit the potential achievements of students affected by mental illness.
  16. That the negative attitudes of others can be a major problem for a student with a mental illness.
  17. The range of mental illness treatments and their effects.

DEALING WITH MENTAL HEALTH CRISES

- Staff should be informed about how to handle mental health crisis situations (e.g. a suicidal person or someone out of contact with reality).
- Staff should be informed about the early signs that someone may be at risk of suicide and what to do if someone is showing these signs.
- Staff should be informed about how to deal with distressed students.
- Staff should be informed about how to deal with students' disruptive or aggressive behaviours.
- Students should be informed about how to handle mental health crisis situations (e.g. a suicidal person or someone out of contact with reality).
- Students should be informed about the early signs that someone may be at risk of suicide and what to do if someone is showing these signs.
- Comprehensive procedures involving security should exist for responding to students who are identified as being in a mental illness crisis to ensure the safety of the individual and of the campus community.
- The institution's response to a mental health crisis should include mechanisms for supporting the campus community in the aftermath of suicide attempts or completions, or other traumatic events, within the limits of confidentiality.

FUNDING

- Adequate funds should be allocated to provide support services to students with a mental illness.
- Institutions should seek funding opportunities that can be used to help develop and enhance support services for students with a mental illness.
- Institutions should consider ways in which they can reduce the financial burden on students, including students with a mental illness, through targeted additional financial support (i.e. scholarships, hardship funds, subsidised accommodation or other support) and by providing assistance to access financial aid or contingency funds.

RESEARCH AND EVALUATION

- The institution should conduct research into the experiences and support needs of students, including those with mental illnesses.
- The institution should conduct research into the barriers to accessing services faced by students with a mental illness.
- The institution's mental health services should be subject to ongoing research and evaluation of their service provision.
- Mental health promotion and training activities carried out within the institution should be formally evaluated.
- The demand for support by students with a mental illness from on-campus services should be monitored regularly in order to ensure that sufficient support services are available.
- Support services should be regularly assessed through a peer-review system to ensure they are of high quality. Institutions should cooperate to support and conduct research on student mental illness.
- The institution should conduct research into new models of service delivery for students with a mental illness.

Although these guidelines are copyright, they can be freely reproduced for non-profit purposes provided the source is acknowledged.

Please cite these guidelines as follows:
Guidelines for tertiary education institutions to facilitate improved educational outcomes for students with a mental illness.
Orygen Youth Health Research Centre, Centre for Youth Mental Health, University of Melbourne; 2011.

Enquiries should be addressed to: Dr Nicola Reavley, Orygen Youth Health Research Centre, Locked Bag 10, Parkville, VIC 3052, Australia. Email: nreavley@unimelb.edu.au

8601_KJOYH_2011

GUIDELINES FOR TERTIARY EDUCATION INSTITUTIONS

THE UNIVERSITY OF MELBOURNE

Orygen YOUTH Health Research Centre

Australian Rotary Health
www.australianrotaryhealth.org.au

Printing funded by the Australian Government Department of Education, Employment and Workplace Relations under the National Disability Coordination Officer Program

GUIDELINES FOR TERTIARY EDUCATION INSTITUTIONS

to facilitate improved educational outcomes for students with a mental illness

These guidelines consist of actions tertiary education institutions can take to facilitate improved educational outcomes for students with a mental illness. They were produced using the Delphi method, which is a systematic way of assessing the consensus of a panel of experts. The actions have been rated as important or essential by expert panels of tertiary education mental health professionals and student consumers. The guidelines will be used to improve the advice to tertiary institutions as they support students with a mental illness.

POLICY

Policy content

- The institution should have a mental health policy covering mental health promotion, mental illness prevention and services for students with a mental illness.
- Disability policies must address the needs of students with a mental illness as well those with physical disabilities.
- Strategic planning in relation to mental health should include objectives, performance indicators, accountabilities and timeframes, so that all involved are clear about what needs to be achieved, who is responsible for achieving it, and by what date.
- The institution should have a policy to ensure that alternative examination and assessment procedures and arrangements are applied consistently across the institution.
- Institution-wide policies and procedures should include confidentiality and disclosure that protect the privacy of an individual with a mental illness.
- Mental health training should be provided to all members of the institution's community (students, staff, faculty) to increase awareness about, recognition of, and the impact of mental illness on study, work and life.
- Institutions should have a mental illness prevention and mental health promotion program.

Policy development and implementation

- The institution's mental health policies should be developed in consultation with outside agencies providing services to students with a mental illness.
- Staff with experience in the field of mental health should play a key role in developing the institution's mental health policy.
- Students with mental illnesses should participate in the development and review of relevant policies, procedures, services and facilities.
- The institution should develop a Disability Action Plan that covers mental illness.
- The mental health policy and its implementation should be driven by senior management in partnership with students with mental illnesses, staff from different areas of the institution, student associations and representatives of outside services.
- There should be a member of the institution's executive who is responsible for mental health policy and its implementation.
- The institution should regularly evaluate the impact of its mental health policy and revise the implementation when necessary.
- The institution's mental health policy should be updated in accordance with legislation, needs of staff, students and stakeholders.

Communicating the policy

- The institution should have a strategy for communicating its mental health policy to staff and students.
- The institution should inform all students that the institution is committed to supporting students with a mental illness.
- Policies and procedures in relation to student complaints, appeals, harassment and disciplinary procedures should cover issues concerning a student's mental illness, and these should be accessible and communicated effectively to all students.
- Effective communication systems (with appropriate consideration for privacy) need to be in place to allow appropriate staff to receive information about the individual needs of students with a mental illness.

SUPPORT SERVICES

Awareness of support services

- Disability services should make all staff and students aware that they provide support to students with mental as well as physical disabilities.
- Counselling services should promote the availability of their support to all students for any difficulties they may be experiencing, including stress, anxiety, personal problems, depression, and performance anxiety.
- The disability office should make all staff aware of the range of services they provide to assist and educate staff supporting students with a mental illness.

What support services should provide

- Support services should develop a mental health promotion strategy, which covers prevention, early identification, stigma reduction, availability and access to services.
- Support services should provide all staff and students with education on mental illness.
- Support services should coordinate with on-campus housing services to educate resident advisors on how to identify and deal with mental illnesses, and what to do in a mental health crisis.
- Support services should proactively promote and publicise information about mental health (including available services) making it widely available (e.g. in student diaries and handbooks, orientation sessions, brochures, global emails, posters around campus and on toilet doors, fact sheets, websites, educational campaigns such as seminars and lectures, at the beginning of lectures, university website, desktop of computers in lecture theatres and computer labs, TV monitors around campus, events in mental health week).
- The institution's support services need to have resources to allow students with urgent or severe problems to be quickly attended to (e.g. have some dedicated crisis appointments).
- The institution's support services need to have a way of prioritising appointments for students with more urgent and severe problems.
- Effective support services should exist for students with a mental illness who are studying through various off-campus enrolments.
- Students with a mental illness should be encouraged by the institution and support services staff to register with these services in order to receive support.
- Information held by support services should be coordinated so that students do not have to repeatedly verify their mental illness.
- Support services should be tailored to be culturally appropriate to different ethnic groups, including international and indigenous students.
- Institutions should, as far as possible, ensure that existing services are responsive to the needs of students with a mental illness rather than create separate service streams specifically for mental illness.
- Support services should make students aware of the extent and limits of confidentiality of these services.
- If the student wishes, support services staff should provide the student with a mental illness with documentation outlining the impact of their mental illness on their study in order to facilitate discussions regarding reasonable adjustments.
- A contact person should be identified who is available to talk to students who have returned to study after periods of mental illness, in order to discuss achievable standards and goals and arrange the necessary academic support.

Accessibility of support services

- Information about how to access mental health services through 'multiple' entry points, either on campus or in the community, should be highly visible (e.g. first level branch on the institution's website).
- Institution enrolment forms should offer students the opportunity to disclose mental or physical illnesses, while explaining to the student that this information will only be used to link them to suitable support services.
- On-campus support services should be easily accessible to all students wherever they are located geographically.
- The institution's support services should adopt an easy access and 'no wrong door' policy to entry for assessment and treatment of mental health problems.
- Support service offices should be easily identifiable.

Relationships with other services

- All staff members in the 'helping network' (including counsellors, disability officers, teaching staff) need to develop good professional relationships to help support the student.
- Where the institution's support services are aware that a student with a mental illness is receiving services from an external mental health agency, they should (with the student's permission) collaborate with that agency to support the student.
- Support services should facilitate communication and coordination with community service providers and be proactive in establishing links with these providers to create networks of support for students with a mental illness.
- If the support services within the institution are not sufficient or appropriate for a student with a mental illness, the institution's services should make arrangements for this student to receive appropriate help at an external service.

OTHER TYPES OF SUPPORT

- A guide should be developed for students with a mental illness on how to get the most out of their studies and time at the institution.
- Student unions and associations should help to reduce the stigma associated with mental illness by being involved with mental health promotion campaigns.
- The institution should offer short courses for students on how to best manage their mental health while fulfilling the student role (i.e. positive coping skills, stress management, study skills).
- Staff involved with vocational and career planning should be trained to have expertise in the area of employment issues of people with mental illnesses.
- Institutions should provide a quiet space where students can go if they feel stressed.

REASONABLE ADJUSTMENTS

- Staff should be provided with information about making reasonable adjustments for assessments.
- Staff should inform students at the beginning of the course about provisions available for reasonable adjustments.
- Staff should encourage students to approach them as soon as possible about any reasonable adjustments they may require.
- If staff give reasonable adjustments to a student, they should record any agreement with the student in writing.
- Staff should periodically review adjustments with the student to assess their effectiveness and make adaptations to changing needs.
- Wherever possible, course content and information should be made available via electronic sources to aid accessibility to students (i.e. audio and video recordings of lectures and tutorials).
- Rather than providing a guaranteed outcome of passing, adjustments and considerations should provide students with a mental illness with an equal opportunity to learn and to demonstrate their knowledge.
- Disability staff who determine reasonable adjustments should do so in collaboration with teaching staff in the relevant discipline.
- The process for getting reasonable adjustments should be as simple as possible and advice should be available to students if needed.

STAFF

Mental illness awareness

The institution should have procedures for making staff aware of the following:

1. What mental illness is.
2. How common mental illnesses are in Australian students.
3. The types of mental illness.
4. The warning signs and symptoms of mental illnesses.
5. The causes of mental illnesses.
6. The range of mental illness treatments and their effects
7. The importance of early identification and intervention for young people who may be developing a mental illness.
8. The things they may notice which might indicate that a student has a mental illness, such as effects on attendance, handing in assignments, displaying unusual behaviours.
9. The benefits for students of disclosing their mental illness to the institution (e.g. to allow access to support services).
10. The fears students may have about disclosing their mental illness (e.g. stigma from others and not wanting to identify as 'crazy').
11. The impact of the symptoms of mental illness on the skills necessary for work and study, such as problems with concentration, memory, decision making and motivation.
12. That the level of support needed by students with a mental illness will fluctuate, as the symptoms of most mental illnesses come and go over time.
13. Measures that can be taken to ensure optimum mental health and prevent mental illness (i.e. self help strategies, seeing a health professional).
14. How they can reduce stressors that increase students' risk of mental illness (e.g. at main assessment times).
15. How they can support their students with a mental illness in ways that promote recovery.
16. The mental health and disability support services available on-campus and in the community.
17. How to refer students who show signs of mental illness to on-campus counselling centres or other mental health resources.
18. That the negative attitudes of others can be a major problem for a student with a mental illness.
19. The myths surrounding mental illness which lead to stigma and limit the potential achievements of students affected by mental illness.
20. The relevant laws and institution policies that affect their interaction with students with a mental illness (e.g. Disability Discrimination Act 1992, Disability Standards for Education 2005).

Mental illness training

- Support services staff should receive appropriate and ongoing professional development and training in relation to mental illnesses.
- Appropriately qualified experts should be involved in mental health training for staff.
- The institution should provide staff with training and information about the following:
    1. The use of non-judgemental listening skills when talking with students about their personal problems.
    2. How to respond when a student discloses a mental illness to them, including which things are supportive and which are unhelpful.
    3. Techniques for promoting motivation and self-esteem in students with mental illnesses.
    4. Curriculum design, development and delivery strategies that facilitate inclusive and effective learning for students with mental illnesses.
    5. Classroom, examination and assignment adjustments that can be made for a student with a mental illness.
- Staff should be encouraged to attend mental health training.
- Senior staff should actively participate in and encourage other staff to do mental health training.
- Staff should make every effort to attend mental health training.
- Staff in student contact roles should be the highest priority for mental health training.
- Staff of student residences who have a pastoral care role should receive mental health training.
- All staff should receive the training necessary to enable them to meet the requirements of students with a mental illness.
- All staff should attend professional development training that continues to provide them with the knowledge and skills to work effectively with students with a mental illness.

Support for staff

- Faculty coordinators and student support staff should encourage discussion among teaching staff about working effectively with students who have a mental illness.
- All staff should have access to an advisor with a higher level of mental health expertise who they can call upon for advice and support when dealing with students with a mental illness.

Communicating with a student with a mental illness

- If a staff member is concerned that a student may have a mental illness, they should express their concern in private to the student, but should leave diagnoses and treatments to the expertise of mental health professionals.
- Staff should interact with students with a mental illness in a manner that maintains respect, dignity, confidentiality and equity.
- When a student discloses that they have personal issues such as a mental illness, confidentiality should be respected unless there is an immediate danger to the person or to others in withholding that information.
- Staff should only ask students with a mental illness for the information about their illness that is relevant to providing support.
- Staff should ask students what, if any, information would need to be shared with other members of staff, or with other students in the class about their mental illness.
- If the student has a mental illness, staff should not make assumptions, but rather ask the student what support, if any, they might need.
- Staff should help students with a mental illness by asking whether they want information about the supports available.
- Staff should explore any challenges or barriers to successful learning with students with a mental illness.
- Staff should always allow sufficient time for discussions with students with a mental illness.
- Staff should be aware of the risk of becoming over-involved with a student with a mental illness, or feeling that it is their responsibility to sort out all the problems.

8601_ter_guidelines.indd 2
21/09/11 3:23 PM
